# Great Expectations: The Role of Rules in Guiding Pro-social Behaviour in Groups with High Versus Low Autistic Traits

**DOI:** 10.1007/s10803-015-2393-x

**Published:** 2015-02-20

**Authors:** Leila Jameel, Karishma Vyas, Giulia Bellesi, Diana Cassell, Shelley Channon

**Affiliations:** 1 Department of Experimental Psychology, University College London (UCL), Bedford Way Building, Gower Street, London, WC1E 6BT UK; 2 Child and Adolescent Mental Health Services, South West London and St George’s Mental Health Trust, London, UK

**Keywords:** Autistic traits, Pro-social behaviour, Empathy, Mentalising, Social rules, Social knowledge

## Abstract

Measuring autistic traits in the general population has proven sensitive for examining cognition. The present study extended this to pro-social behaviour, investigating the influence of expectations to help others. A novel task describing characters in need of help was administered to students scoring high versus low on the Autism-Spectrum Quotient. Scenarios had two variants, describing either a ‘clear-cut’ or ‘ambiguous’ social rule. Participants with high versus low autistic traits were less pro-social and sympathetic overall towards the characters. The groups’ ratings of characters’ expectations were comparable, but those with high autistic traits provided more rule-based rationales in the clear-cut condition. This pattern of relatively intact knowledge in the context of reduced pro-social behaviour has implications for social skill training programmes.

## Introduction

Successful social functioning entails processing and responding sensitively to subtle and complex information provided by the social world. Whilst there is a wealth of literature exploring cognitive accounts of autism spectrum disorder (ASD), very little work has examined how these translate into everyday social functioning and impact on specific aspects of social interaction, in particular pro-social behaviour such as helping and sharing with others, volunteering time or donating money. Two studies examining charitable giving found that adults with ASD donated less than matched controls (Lin et al. 2012; Izuma et al. 2011). Parental reports indicated that children with ASD tend to display significantly less pro-social behaviour compared to typically developing children, e.g. being kind and considerate to others, sharing or offering practical help (Meyer et al. 2006; Allik et al. 2006). Several small-scale interventions using social stories have attempted to promote pro-social behaviour in children with ASD (e.g. see Crozier and Tincani 2007; Leaf et al. 2009). Recent work has examined the use of oxytocin, which is thought to play a role in regulating and promoting social behaviour, in individuals with ASD, and this seems to show some promise (e.g. see Andari et al. 2010).

In the light of the established social and emotional deficits associated with ASD, it is important to understand what predicates pro-social behaviour, and how factors in the social environment might facilitate or inhibit this. Pro-social behaviour is thought to be driven by empathy (Eisenberg 2007; Minio-Paluello et al. 2009), via ‘self’- or ‘other’-oriented processes (Schaller and Cialdini 1988). Vicariously invoked feelings of distress and increased physiological arousal when witnessing someone in need may result in a desire to alleviate the pain shared by the onlooker, thus stimulating a response to help. The resulting action is thus self-oriented and motivated by a need to reduce the vicarious empathic arousal experienced. On the other hand, pro-social behaviour may be driven by an intuitive understanding of the other’s thoughts, feeling and needs (Jameel et al. 2014).

The self- versus other-orientated routes for motivating pro-social behaviour can be viewed as analogous to the separable ‘emotional’ and ‘cognitive’ components that contribute towards the experience of empathy. ‘Emotional’ empathy refers to the ability to resonate with and share another’s emotional state. By contrast, ‘cognitive’ empathy, or mentalising (also synonymous with theory of mind or perspective-taking), describes the ability to infer others’ mental states and thus what they might be thinking or feeling in any given situation (for a discussion of cognitive vs emotional ‘fine cuts’ see: Blair 2008). Mentalising is thought to be impaired in individuals with ASD, with difficulties in interpreting others’ intentions, thoughts and feelings, whereas most evidence suggests that ability to empathise with others’ emotional states is intact. However, upon close inspection the literature reveals a slightly messier picture. Some studies examining emotional empathy have found impairment in those with ASD (Humphreys et al. 2007) and others have not (Adolphs et al. 2001). Where there is evidence for intact emotional empathy, this is thought to reflect some capacity of those with ASD to resonate emotionally with others, but via a self-stance (Frith and de Vignemont 2005; Minio-Paluello et al. 2009). However, whilst the cognitive and emotional components of empathy are thought to be dissociable in principle, they are likely to be used in concert in everyday life.

It has been suggested that individuals with high-functioning ASD sometimes rely on compensatory strategies, such as the application of learned social rules to alleviate mentalising deficits (Hill and Frith 2003). This is supported by neuroimaging evidence demonstrating that during online mentalising, high-functioning individuals with ASD show reduced activation in brain regions typically associated with this type of activity, even when correct mental state attributions are made (Happe et al. 1996). Conversely, individuals with high-functioning ASD tend to show greater activation in areas typically associated with more general problem-solving abilities (Happe et al. 1996). Reliance on compensatory mechanisms such as drawing on previously acquired social knowledge may disguise social difficulties to an extent, but clumsy and inflexible patterns of social behaviour may occur in more complex, unpredictable social circumstances.

Clinical accounts tend to support the notion that high-functioning individuals with ASD draw upon compensatory mechanisms to navigate the social world. Müller et al. (2008) individually interviewed adults with ASD to discuss their social and communication challenges. Their descriptions included difficulties with ‘chit-chat’ conversations that tend not to follow predictable sets of rules, and problems in following unstructured dialogue that require improvised responses. References were also made to actively learning how to behave socially via observation of others’ social interactions. Whilst appropriate social behaviour is thought to involve the acquisition of knowledge about social roles, norms, and scripts, it also requires flexible application of these resources to navigate novel scenarios accurately (Riggio and Reichard 2008).

Evidence from real-life-type tasks suggests that high-functioning individuals with ASD may show proficiency in simple structured social tasks, but difficulties with more advanced and naturalistic tasks. For instance, when asked to make judgments about subtle social behaviours (e.g. faux-pas or deception), individuals with high-functioning ASD may provide correct answers, but the reasoning behind their judgments is often inaccurate or inappropriate (Bowler 1992; Moran et al. 2011). Moreover, in real-life-type problem solving tasks, high-functioning participants with ASD have been found to display difficulty in generating problem solutions, but not in selecting from alternatives presented to them (Channon et al. 2001, 2014). Participants with ASD were thus able to recognise the best solution from a selection of options (i.e. low task demand), but not to produce high quality solutions spontaneously (i.e. high task demand).

Studies of individuals with high autistic trait scores drawn from the general population has suggested a pattern of social, cognitive and emotional features similar to, but less severe than that typically observed in ASD. For example, Gökçen et al. (2014) found that higher numbers of autistic traits were associated with lower self-rated levels of emotional perception and expression, poorer performance on a classic task involving predicting characters’ emotional states from pictures of their eyes, and impairment on a task examining cognitive flexibility.

A recent study (Jameel et al. 2014) examined everyday pro-social performance in students scoring high versus low on a measure of autistic traits (Autism-Spectrum Quotient (AQ); Baron-Cohen et al. 2001). Participants were presented with everyday situations involving a character in need of their help. Each scenario thus involved a difficult social judgment with respect to balancing the needs of the character against the participant’s own interests. For instance, if someone sees a man fall over heavily in front of them, do they stop to help, even if it means being late to work? Those scoring high on the AQ were found to behave less pro-socially than their low AQ counterparts, both when asked to generate pro-social courses of action, and to select them from alternatives. Participants were also asked to give self- versus other-satisfaction ratings corresponding to three different courses of helping actions, ranging from low to high pro-social value (i.e. carry on walking, stop briefly to help the man up, or stop to help the man up and see what additional assistance he may require). Contrary to predictions, those with high AQ scores gave similar estimates of the characters’ sense of satisfaction, but reported less personal satisfaction than those with low AQ scores for going ‘above and beyond’ to help others at their own expense.

Lower levels of pro-social behaviour might be linked to limited perception of societal expectations, a reduced sense of pressure to comply with such expectations, and lower rewards for helping others. The present study sought to explore this by examining the role of societal expectations and sympathy for others’ needs in guiding pro-social decision making. Participants with high or low AQ scores were presented with a new set of scenarios featuring a character in need. Each scenario had two variant endings: a clear-cut versus ambiguous social rule. In the ‘clear-cut’ condition there was a strong social rule guiding the participants to behave pro-socially (i.e. offer to give up your seat to an elderly woman walking with a stick). In the ‘ambiguous’ condition the social rule was weaker (i.e. offer to give up your seat to a young woman carrying a heavy parcel).

Participants were asked to reason about why someone might act pro-socially in the situation, and were also asked to rate the characters’ expectations of help, their own likelihood to help, and their sympathy for the characters in need. It was predicted that the high AQ group would generate rationales for pro-social behaviour that relied upon social rules, rather than engaging with the individual perspectives of the characters, at least in the clear-cut condition where there was a readily available social rule. However, on the lower-demand measure of rating characters’ expectations of help, it was postulated that the high AQ group might not differ from the low AQ group, at least for the clear-cut condition. It was predicted that those with high AQ traits would be slower to produce responses, at least in the ambiguous condition when less salient cues guiding pro-social behaviour were provided. Although little work has examined this, it seemed probable that sympathy ratings and likelihood of helping would be reduced in the high AQ group, especially in the ambiguous condition where social rules were less clear. However, it was also considered possible that the high AQ group might differentiate more than the low AQ group between the clear-cut and ambiguous conditions in their sympathy ratings and likelihood of helping, showing more ‘black and white’ thinking consistent with a rigid reliance on social rules. In support of this prediction, some previous work with individuals with ASD reported that they showed heightened sensitivity to ‘good’ versus ‘poor’ justifications for wrongdoing (Channon et al. 2010), and greater differentiation between intentional and unintentional actions when assigning blame (Channon et al. 2011).

## Methods

### Screening Phase

#### The Autism-Spectrum Quotient

The Autism-Spectrum Quotient (AQ; Baron-Cohen et al. 2001) is a brief, self-report questionnaire with good internal consistency, construct validity and test–retest reliability, which measures personality traits associated with the autistic spectrum in adults of typical intelligence. It uses a four point Likert scale and yields total AQ trait scores ranging from 0 to 50, with higher scores indicating higher numbers of autistic traits. This measure was not designed as a diagnostic tool, but rather for the identification of traits and behaviours related to the autistic profile, containing 50 statements covering five different aspects of autistic symptomatology: social skill, attention switching, attention to detail, communication and imagination.

#### Participants and Procedure

Ethical approval was obtained from the UCL Research Ethics Committee. An opportunistic sample of 645 full-time university students (41.39 % female) who were fluent in English and aged 18 or over (mean age 20 years) was recruited for the screening phase of the study. All participants provided informed consent before completing the AQ (Baron-Cohen et al. 2001). Participants were entered into a prize draw and informed that they might be invited to take part in the second phase of the study, for which they would be paid. Total AQ scores were calculated for the whole sample. AQ traits are more common in males than in females (Baron-Cohen et al. 2001), thus in order to form gender balanced high AQ and low AQ groups for the experimental phase of the study participants within the highest-scoring and lowest-scoring 10 % of males, and highest-scoring and lowest-scoring 10 % of females, were selected. Participants were contacted via email or telephone and invited to take part in the second stage of the study.

### Experimental Phase

#### Participants and Procedure

20 (10 female, 10 male) individuals from the lower range and 21 (11 male, 10 female) individuals from the upper range took part in the experimental phase of the study, forming two groups of low and high AQ participants. AQ scores ranged from 2 to 9 in the low AQ group (2–9 for male and 4–8 for female participants), and 26–46 in the high AQ group (28–37 for male and 26–46 for female participants). A *t* test confirmed that AQ scores differed significantly between groups, *t*(1,39) = 23.23, *p* < .001; mean AQ scores were 30.52 (SD = 4.40) and 6.15 (SD = 1.66) for the high and low AQ groups respectively. The groups did not differ significantly in age, *t*(1,39) = .064, *p* = .950; mean age was 21.11 (SD = 2.62) and 21.06 (SD = 2.57) for the high and low groups respectively.

All participants were tested individually, and provided written informed consent before completing the ‘Social Expectations’ task. Participants were also asked to complete a brief health-screening questionnaire that asked about any serious accidents or illnesses, psychological or emotional difficulties; in practice no exclusions were required. Participants were paid for their time and efforts.

#### The “Social Expectations” Task

The “Social Expectations Task” was designed to examine pro-social behaviour in relation to some of the unwritten social rules that govern everyday interactions, comparing scenarios based on both clear-cut and ambiguous rules. A range of scenarios were devised and piloted, in order to refine the items and develop the scoring systems. The final task consisted of 10 hypothetical scenarios involving the participant and an unfamiliar character. The scenarios consisted of everyday social situations where the participant had the opportunity to engage in pro-social behaviour aiding the character, in line with a social rule. Scenarios were designed such that it was clear the participant was the *only* individual who could aid the character, and that engaging in the pro-social behaviour would be inconvenient to the participant (e.g. offering to give up your seat and stand for someone). The character was male in half the scenarios, and female in the other half, and the social context varied across scenarios to reflect a range of natural situations. To control for order effects, two different scenario orders were created and counterbalanced within each group.

Each scenario stem had two endings, manipulating the strength of the social rule guiding pro-social behaviour in the situation. ‘Clear-cut’ endings implied a strong social rule (e.g. “she is elderly and walking with a stick”), cueing an appropriate response (i.e. you should offer to give up your seat). ‘Ambiguous’ endings still referred to a character that would benefit from help, but did not rely on such strongly endorsed social rules (e.g. “she is a young adult and is carrying a large parcel”). For each scenario ending participants were asked to give three ratings, indicating how likely they would be to behave pro-socially, how sympathetic they would feel towards the character, and how strongly the character expected their help. Participants were also asked to generate verbal responses explaining why they might choose to help the character in the given situation.

All participants first read a sheet of instructions about the task. This explained that they would see short scenarios about everyday situations and would respond verbally to questions, supplying either ratings or free responses. Participants were requested to answer as quickly and truthfully as possible. The scenarios were presented on paper, and participants were taken through an example before completing the 10 experimental items. Scenarios and questions were presented in separate booklets such that relevant scenarios remained on display throughout task performance, in order to minimise any memory demands. Each scenario was followed by four questions. Participants were first asked to rate how likely they would be to help the characters, and then to rate how sympathetic they felt towards the characters. Participants were then asked to indicate the strength of the characters’ expectations for help, and finally to provide a rationale explaining why they might offer to help the character. Response times for rationale production were recorded using a stopwatch. Total response time was calculated by summing the speed of response time across all 10 scenarios.

### Example Scenario

You are sitting in a crowded waiting room with a small bag, waiting for a delayed train. All the other seats are taken by passengers with lots of luggage. A woman enters the waiting room looking for a seat.

*Clear*-*cut Ending:* She is elderly and walking with a stick
*Ambiguous Ending:* She is a young adult and is carrying a large parcel

### Questions for Each Scenario

*Likelihood of Pro*-*social Behaviour Ratings:* How likely is it that you would offer her your seat?
On a scale of 1–10: 1 = not at all likely, 10 = very likely
*Sympathy for Character Ratings:* How sympathetic do you feel towards her?
On a scale of 1–10: 1 = not at all sympathetic, 10 = very sympathetic
*Strength of Character Expectation Ratings:* How much do you think she expects you to offer her your seat?
On a scale of 1–10: 1 = not at all, 10 = very much
*Verbal Rationales of Societal Expectation Understanding:* Why might you offer her your seat?

### Scoring

#### Likelihood of Pro-social Behaviour Ratings

For each scenario, participants rated the likelihood of offering to help the characters on a scale of 1–10, where higher scores indicated greater pro-social behaviour. Ratings were then summed across all 10 scenarios to create a total score for each condition (range 10–100), creating 2 scores: (1) clear-cut rule pro-social behaviour rating, (2) ambiguous rule pro-social behaviour rating.

#### Sympathy for Character Ratings

For each scenario, participants rated the degree of sympathy they experienced for the characters on a scale of 1–10, where higher scores indicated greater sympathy. Ratings were then summed across all 10 scenarios to create a total score for each condition (range 10–100), creating 2 scores: (1) clear-cut rule sympathy rating, (2) ambiguous rule sympathy rating.

#### Strength of Character Expectation Ratings

For each scenario, participants rated how much they thought the characters’ expected their help on a scale of 1–10, where higher scores indicated a greater expectation to help. Ratings were then summed across all 10 scenarios to create a total score for each condition (range 10–100), creating 2 scores: (1) clear-cut rule character expectation for help rating, (2) ambiguous rule character expectation for help rating.

#### Verbal Rationales of Societal Expectation Understanding

Verbal responses were categorised according to two dimensions: rule-based or person-based rationales. The person-based rationales reflected responses that referred to the characters’ needs, and/or conveyed a sense of self-sacrifice on the part of the participants, in order to meet the characters’ needs. Rule-based rationales reflected responses that made explicit reference to a social rule guiding an expectation to help, or implied a social rule by simply referring to the facts of the scenario. Scoring of the example scenario is shown below in Table 1. Responses could only score for one of the two dimensions; if both dimensions were met then the best answer was taken, and thus participants scored for person-based rationales.

**Table 1:** Scoring of example scenario from the ‘Social Expectations’ task

*Description of criteria*
**Person-based rationales**
A response that either referred to the characters’ needs, and/or conveyed a sense of self-sacrifice on the participants’ part in order to meet the characters’ needs
**Rule-based rationales**
A response that made explicit reference to a social rule guiding an expectation to help, or implied a social rule by simply referring to the facts of the scenario
**Example responses:**
***Clear*** **-** ***cut ending:*** “She is elderly and walking with a stick”
**Person-based rationale examples**
e.g. “I would feel sorry for her and it would be difficult for her to stand in a crowded waiting room”
e.g. “She needs it more than I do”
**Rule-based rationale examples**
e.g. “You should always offer your seat to women, elderly and the disabled”
e.g. “She is walking with a stick”
***Ambiguous ending:*** “She is a young adult and is carrying a large parcel”
**Person-based rationale examples**
e.g. “She must be feeling very tired”
e.g. “I think she needs it more than I do because she is carrying a large parcel”
**Rule-based rationale examples**
e.g. “To be polite”
e.g. “It is common courtesy to offer your seat”

The responses were classified by one blind independent rater, and one rater who was not blind to group membership. There was an inter-rater agreement rate of 90.73 %; all disagreements were resolved between the raters via discussion. Once all responses had been classified and disagreements resolved, participants’ scores were summed across all 10 scenarios, and the percentage of person-based versus rule-based responses was calculated for both the clear-cut and ambiguous conditions.

## Results

### Data Analysis

Means and standard deviations (SD) for each of the measures are presented below in Table 2. A significance level of .05 was adopted, with adjustment for post hoc *t* tests (.05/2) to control for multiple comparisons.

**Table 2:** Mean percentage scores and standard deviations for all measures for the ‘Social Expectations’ task

	Low AQ group (N = 20) M (SD)	High AQ group (N = 21) M (SD)	Significance		Effect Size
*Likelihood* (*%*)			Condition	**	
			Gp	*	
			Gp × condition	NS	
Clear-cut	86.95 (9.26)	78.42 (9.68)	–		0.90
Ambiguous	55.10 (10.16)	48.05 (12.73)	–		0.61
*Sympathy* (*%*)			Condition	**	
			Gp	**	
			Gp × condition	NS	
Clear-cut	80.25 (9.25)	68.81 (13.09)	–		1.01
Ambiguous	43.85 (9.55)	35.00 (12.62)	–		0.79
*Strength of expectation* (*%*)			Condition	**	
			Gp	NS	
			Gp × condition	NS	
Clear-cut	76.05 (9.01)	73.24 (2.17)	–		0.43
Ambiguous	54.15 (8.77)	50.76 (11.09)	–		0.34
*Verbal rationale classification* (*%*)			Condition	NS	
			Gp	NS	
			Gp × condition	**	
Clear-cut					
Rule	33.00 (18.38)	49.05 (25.08)	025**		0.73
Person	67.00 (18.38)	50.95 (25.08)	0.73		0.73
Ambiguous					
Rule	40.5 (20.64)	39.05 (13.38)	–		0.08
Person	59.5 (20.64)	60.95 (13.38)	–		0.08
*Verbal rationale response time* (*s*)					
			Condition	NS	
			Gp	NS	
			Gp × condition	**	
Clear-cut	87.35 (24.09)	90.76 (31.61)	–		0.12
Ambiguous	81.30 (20.75)	101.71 (42.53)	–		0.61

** Significant at *p* = .01; ** Significant at *p* = .025

### Social Expectations Task

#### Likelihood of Pro-social Behaviour Ratings

A repeated measures 2 × 2 ANOVA was conducted to examine ratings for likelihood of behaving pro-socially for all scenarios. There was one between-participant factor (AQ group: high vs low AQ) and one within-participant factor (ambiguity of social rule: clear-cut vs ambiguous). There were significant main effects of condition, *F*(1,39) = 491.87, *p* = .0001, and of group, *F*(1,39) = 6.79, *p* = .013. The condition by group interaction was not significant *F*(1,39) = .274, *p* = .604.

From inspection of the mean scores presented in Table 2, it is clear that all participants were more likely to behave pro-socially when the social rule was clear-cut versus ambiguous. This is in line with the prediction that a clear-cut rule would enhance the characters’ expectation for help, and thus also the likelihood of complying with it. The high AQ group was less pro-social overall; the lack of condition of social rule by group interaction suggests that the groups were not however differentially affected by the strength of the social rule.

#### Sympathy for Character Ratings

A repeated measures 2 × 2 ANOVA was also conducted to examine ratings for sympathy for all scenarios. There were significant main effects of condition, *F*(1,39) = 356.63, *p* = .0001, and group, *F*(1,39) = 11.4, *p* = .002. However, the condition by group interaction was not significant *F*(1,39) = .486, *p* = .490.

The mean scores suggested that all participants were more sympathetic when the rule was clear-cut versus ambiguous. The high AQ participants were less sympathetic towards characters overall; the lack of condition of social rule by group interaction suggests that the groups were not however differentially affected by the strength of the social rule.

#### Strength of Character Expectation Ratings

A repeated measures 2 × 2 ANOVA was also conducted to examine ratings for the strength of the characters’ expectation for help across all scenarios. There was a significant main effect of condition, *F*(1,39) = 175.41, *p* = .0001. The main effect of group, *F*(1,39) = 1.24, *p* = .272, and the condition by group interaction, were not significant *F*(1,39) = .30, *p* = .864.

This confirms that the social rule manipulation operated as intended; all participants identified a stronger character expectation to help when the rule was clear-cut versus ambiguous. The high AQ group rated the scenarios similarly to the low AQ group identifying a stronger character expectation for help in the clear-cut versus ambiguous condition.

#### Verbal Rationales of Societal Expectation Understanding

Finally the high and low AQ groups were compared for their verbal responses outlining why one might choose to help the character in each scenario. A repeated measures 2 × 2 ANOVA was conducted to examine the percentage of rationales classified as rule-based versus person-based for the two conditions. The main effect of condition was not significant *F*(1,39) = .114, *p* = .738, nor was the main effect of group *F*(1,39) = 2.161, *p* = .150. However, there was a significant condition by group interaction, *F*(1,39) = 5.57, *p* = .023. Post-hoc *t* tests, using a strict significance level, showed that the high AQ group used significantly more rule-based versus person-based rationales than the low AQ group in the clear-cut condition, *t* (1,39) = 2.327, *p* = .025; there was no significant group difference in the ambiguous condition, *t*(1,39) = .269, *p* = .790.

Total response time for rationale production was also examined by summing the speed of response time across all 10 scenarios and comparing the group averages. A repeated measure 2 × 2 ANOVA was conducted to examine the speed of response time in the clear-cut versus ambiguous condition. The main effect of condition was not significant *F*(1,39) = .635, *p* = .431, neither was the main effect of group *F*(1,39) = 1.672, *p* = .204. As with the rationale classification measure, there was a significant condition by group interaction, *F*(1,39) = 7.632, *p* = .009. Post-hoc *t* tests, using a strict significance level, did not reach significance. However, inspection of the mean scores indicated a tendency for the high AQ group to be selectively slower in the ambiguous, *t* (1,39) = 1.967, *p* = .060, vs clear-cut condition, *t*(1,39) = .387, *p* = .701.

## Discussion

The present study examined the role of social rules in guiding pro-social behaviour, and how this might be influenced by autistic traits. A scenario-based task was developed describing everyday situations in which a character required help. Each scenario had two conditions: it ended with either a clear-cut or an ambiguous social rule guiding pro-social behaviour. Participants’ likelihood of complying with these societal expectations and their sympathy for the character was assessed using rating scales. Their ‘understanding’ of the social expectation to help was also assessed by two measures. Firstly, they indicated the strength of the characters’ expectations of help using a rating scale. Secondly, their justification for why one would help was assessed via the generation of verbal rationales.

The key finding with respect to pro-social behaviour was that, as expected, the high AQ participants were less likely to behave pro-socially overall, but the groups were not differentially affected by the strength of social rule manipulation. Thus, all participants were more compliant with the expectation to help characters in the clear-cut versus ambiguous condition. For instance, in the example scenario, participants were much more likely to help when the character was elderly and walking with a stick, but far less likely to help when she was young and carrying a heavy parcel; the high AQ group gave lower likelihood of helping ratings than the low AQ group across conditions. With respect to the sympathy ratings, all participants were more sympathetic in the clear-cut versus ambiguous condition. The high AQ group was thus less likely to feel sympathy for the characters, regardless of the clarity of the social rule.

Did those with high AQ traits understand the societal expectations inherent in the scenarios? One way of estimating these societal expectations was by asking participants to rate the extent to which the characters expected help. Interestingly, the groups did not differ in this; the high AQ group was equally able to identify the stronger expectation to behave pro-socially in the clear-cut versus ambiguous condition. However, when asked to complete the more demanding task of providing verbal rationales outlining why one might behave pro-socially, the picture was more complex. In the clear-cut condition the low AQ group used more person-based rationales (e.g. she needs the seat more than me) than rule-based rationales reflecting societal expectations (e.g. you should always give up your seat for the elderly), whereas the high AQ group used these equally. In the ambiguous condition, both groups used slightly more person-based than rule-based rationales. Inspection of the rationale production response times revealed a condition by group interaction suggesting that the high AQ group was selectively slower for the ambiguous versus clear-cut condition when producing rationales, although this did not hold as significant when post hoc t-tests were conducted.

The findings of the present study corroborate those of Jameel et al. (2014), providing further evidence of reduced pro-social behaviour in individuals with high numbers of autistic traits. The two studies taken together also reveal some hint of preserved social knowledge in the high AQ group, but differences between groups in the socio-emotional processes thought to motivate pro-social behaviour. Various theoretical accounts associated with ASD may be relevant for explaining the pattern of findings reported in those with high AQ traits, in particular the role of emotional empathy, mentalising, and the potential influence of social knowledge and learning. It is also possible that non-social models such as impaired executive functioning (Hill 2004) or weak central coherence (Frith 1989, 2003) make a contribution, although they are not discussed in detail here.

### Impaired Emotional Empathy?

Since both emotional and cognitive components of empathy are thought to play a role in motivating pro-social behaviour (Eisenberg 2007), this theoretical account could explain the decreased pro-social behaviour displayed by the high AQ group. With regard to ASD, although it has been posited that this is associated with impaired mentalising but intact emotional empathy (Blair 2008), there is some conflict in the literature exploring this. Some studies examining the recognition of, and response to, affective expressions have reported impairment in ASD (Humphreys et al. 2007) and others have not (Adolphs et al. 2001). This inconsistent picture may reflect the differing intellectual capabilities of individuals with ASD and/or a variety of task demands (Blair 2008).

It has also been suggested that those with ASD may possess limited capacity to resonate emotionally with others via a self-focused stance, mediated by a heightened sense of egocentricity, at the expense of the ability to take an allocentric (other-focused) stance (Frith and de Vignemont 2005; Minio-Paluello et al. 2009). With respect to the present AQ study, it is possible that an imbalance in the priorities placed on self versus other needs resulted in less motivation to behave pro-socially, regardless of whether the high AQ group could correctly identify and understand the characters’ needs. The high AQ participants may therefore have focused on themselves and prioritised their own interests at the expense of helping others, and experiencing reduced resonance with the characters’ points of view may have compounded this.

However, impairment in emotional empathy could not account for the greater tendency of the high AQ group to use rule-based rationales in the clear-cut condition when reasoning about why one should behave pro-socially, nor the differences observed in the speed of rationale production. Impairment in emotional empathy in the context of intact mentalising abilities would not be expected to affect the high AQ group’s capacity to understand how they should behave and why; rather, it should selectively influence their actual behaviour. Nonetheless, a possible contribution of emotional empathy cannot be dismissed, since it is difficult to disentangle the relative contributions of this versus mentalising in relation to measures designed to explore everyday social behaviour.

### Impaired Mentalising?

A mentalising explanation would contend that other-oriented pro-social behaviour is dependent upon appreciating others’ perspectives, and acting accordingly; failure to do so may have reduced the participants’ incentive to help the characters, resulting in reduced compliance with the societal expectation to behave pro-socially. This explanation is consistent with some evidence in studies of those with high autistic traits suggesting differences on tasks tapping mentalising including social attention (Freeth et al. 2013) and poorer performance on false belief tasks (Best et al. 2008).

The sympathy ratings may provide the most direct measure of mentalising. Sympathy refers to feelings of concern about the welfare of others, and is thought to play a motivating role in pro-social behaviour via other-oriented processes (Decety and Michalska 2010). Whilst sympathy and empathy are often conflated, they are in fact distinct concepts; the experience of sympathy is said to be dependent upon the mentalistic ability to apprehend another’s mental state, but it does not necessarily require a vicarious emotional experience (Decety and Chaminade 2003). On this basis, sympathising with characters in the social expectations task requires participants to put themselves in others’ shoes and imagine how they would feel in such a situation, and is potentially mediated by mentalising abilities. Lower sympathy ratings by the high AQ group are therefore consistent with the evidence of impaired mentalising in those with ASD.

Some previous work has reported ‘black and white’ sympathy ratings in those with ASD, with heightened sensitivity to ‘good’ versus ‘poor’ justifications for wrongdoing (Channon et al. 2010), and greater differentiation between intentional and unintentional actions when assigning blame (Channon et al. 2011). No evidence of such ‘black and white’ thinking was found in the present AQ study, since sympathy ratings were lower overall in the high versus low AQ group, and the groups were not differentially affected by the strength of the social rule. However, the nature of the present study is different to those previously used, since in the Channon et al. tasks, lack of sympathy in the group with ASD related to characters acting for reasons that generally contravene societal, and indeed legal, expectations (e.g. drunk driving after a party, or intentionally giving a spouse an overdose of their medication). By contrast, in the present AQ study, the characters were all deserving of help and thus sympathy, regardless of the condition.

### The Role of Social Knowledge and Learning

One potential caveat for an interpretation consistent with a mentalising deficit is that those with high AQ traits did not differ from the low trait group on some measures assessing participants’ understanding of characters’ expectations for help. At first glance it might appear that a mentalistic appraisal is required to grasp the characters’ expectations. However, routes other than mentalising may also lead to similar judgments of the characters’ expectations, such as reliance on knowledge of societal norms. More broadly, it has been suggested that individuals with ASD may not rely on intuitive socio-emotional processes for solving social and moral dilemmas (Greene and Haidt 2002). Rather, it may be a more laborious process in which individuals with ASD learn about and apply social rules, especially when these are readily available.

Thus, in the clear-cut task condition of the current study where the social rules were more salient, the high AQ group appeared to draw upon these rules by providing more rule-based and fewer person-based justifications for the reasons surrounding why one should act pro-socially. On the other hand, when salient rules were not available in the ambiguous condition, the high AQ group was able to produce person-based rationales as often as the low AQ group. This may indicate a stylistic preference for rule-based reasoning over reliance on mentalistic processes, which may be more effortful for them. This interpretation is also supported by the finding that those with high AQ traits tended to be slower to produce rationales only in the ambiguous condition. A lack of salient social rules underpinning the scenarios may have forced them to exert more effort and employ person-based rationale, making heavier demands on mentalising ability.

This is also consistent with previous literature indicating that individuals with ASD might provide correct answers, but that the reasoning behind their judgments is often more limited (Moran et al. 2011). For instance, children with ASD have been found to show accurate moral judgements (Grant et al. 2005; Leslie et al. 2006), and intact understanding of transgressions involving breaking social versus moral rules (Blair 1996), but to display difficulties in justifying their choices. Zalla et al. (2009) similarly found that adults with ASD were able to use compensatory strategies to carry out social judgments regarding faux-pax, but failed to justify their responses adequately.

Interpreting the findings of the present AQ study in the light of previous relevant work in ASD, it appears that individuals with high numbers of autistic traits may show some preservation of social judgement and assimilate the characters’ expectations (a mentalistic demand), by seemingly exploiting their knowledge of rules regarding societal norms. Further evidence supporting the use of compensatory mechanisms within the autistic spectrum can be derived from a recent neuroimaging study that asked why some children with ASD pass classic mentalising tasks, and others do not (White et al. 2014). They subdivided ASD participants into groups who either failed or passed such tasks, and compared their brain activation patterns with those of typically developing children during an online mentalising task. Regardless of whether they belonged to the ‘fail’ or ‘pass’ groups, participants with ASD showed differences in brain regions associated with mentalising in comparison to typically developing children. This suggests that even individuals with ASD who pass such tasks may be doing so via an alternative route.

In the present AQ study, reliance on compensatory mechanisms such as social knowledge may have circumvented the need to employ mentalistic processes when estimating the characters’ expectations. However, this may be at the expense of resonating with someone emotionally, and hence this may explain why the high AQ group reported less sympathy with the characters, and were less likely to help them. Both mentalisitic abilities and emotional empathy play a key role in facilitating socially sensitive behaviour, and it is likely that healthy individuals use both social knowledge stores and socio-emotional processes to assess social scenarios and respond appropriately. In order to make good use of social rules to deal with complex social stimuli in a range of contexts, flexibility is required to learn about the contingencies for applying rules appropriately to different conditions (Nelson and Guyer 2011; Bunge 2004). This is likely to draw upon executive skills, which are thought to be impaired in individuals diagnosed with ASD (see Hill 2004 for a review). From a developmental perspective, as children become adolescents, and in turn adults, the complexity of the social environments they navigate increases dramatically. In typically developing children, this should be supported by gradually enhanced social knowledge, as a result of exposure to more challenging and ambiguous social environments. However, in children and adolescents with ASD, who tend to avoid social engagement (Richer 1976), and/or experience the social world in an ‘atypical’ fashion (Hughes and Leekam 2004), such learning may be deficient. Thus, the acquisition of social rule knowledge and its use may be further protracted or stunted in those with ASD. With respect to the current study, this could account for the high AQ group’s failure to put their relatively preserved knowledge of social rules into practice by choosing to help the characters.

### Implications and Applications for Everyday Social Functioning

The present study examined individuals drawn from the general population with high AQ traits and did not require a clinical diagnosis of ASD. One might expect those with high AQ traits to show a muted, albeit similar, pattern of social performance to those with a clinical diagnosis of ASD. Although there is no evidence of a bimodal distribution separating out clinical and non-clinical levels of impairments (Skuse et al. 2005), a clear dose–response relationship for degree of autistic traits has yet to be established. Whilst the pattern of findings from the present study is broadly consistent with literature examining social functioning in individuals diagnosed with ASD versus neurotypical controls, it would nevertheless be important to extend these measures to a clinical sample before clinical applications could be developed from these findings. This could also help to establish the validity of the assumption that there is a continuum of social ability relating to the number and severity of autistic traits, regardless of a clinical threshold.

Social difficulties such as problems in forming or maintaining interpersonal relationships or engaging in inappropriate behaviour are often central to the everyday struggles that individuals with ASD face (Troisi 2008; Crespi and Badcock 2008). In order to design effective interventions, such as social skill training, it is important to have a detailed understanding of how individuals’ symptoms may interact with the environment and disrupt everyday functioning. At present, this level of understanding is very limited, with most work focusing on either abstract laboratory based tasks or clinical reports. Tasks such as the current one provide a rich source of material for understanding of the nature of everyday difficulties that those with high AQ traits, or possibly a diagnosis of ASD, may face. It could potentially be used in clinical settings to identify factors that may facilitate or impinge upon successful everyday social functioning, and to better inform interventions (Channon et al. 2014). The findings of the present study with respect to reduced pro-social behaviour in those with high AQ traits, highlight the need for social skills training to embrace both expanding individuals’ knowledge of social rules (e.g. you should give up your seat to someone elderly), and to target the flexible application of social rules to novel situations. In individuals diagnosed with ASD an overreliance on social rules, without a deeper understanding of the social implications or caveats, may lead to awkward and inflexible patterns of social behaviour. Compensatory cognitive strategies can be useful in the face of reduced or absent capacity to access appropriate socio-emotional processing. However, they must be combined with enhanced social motivation; otherwise, individuals may be aware of the rules, but fail to act on them, as appears to have been the case in the present study. Social motivation is an area of research that has been neglected in ASD, and which requires further exploration. For instance, it would be interesting to study how rewards are processed by individuals with high AQ traits or ASD, and which kind of rewards might facilitate appropriate behaviour.

A potential limitation of the study concerns the validity and sensitivity of measures such as the Social Expectations task that attempt to tap everyday social behaviour. The strength of tasks such as this is that they use real-life-type materials to investigate performance. Real-life-type materials have advantages over more traditional abstract laboratory tasks, since they are more likely to draw upon both social and practical knowledge acquired both directly and indirectly through life experience, in addition to intellectual skills. Despite these advantages, however, any task that is carried out under experimental conditions with structured cues that constrain and prompt performance in a manner that can be easily measured and interpreted, has limitations with respect to real world validity. Such cues are often lacking in everyday life and their presence in experimental tests may thus lead to a lack of correspondence with real world performance and an underestimation of social difficulties outside of the laboratory (Channon et al. 2001). This is particularly pertinent when assessing individuals who share characteristics of ASD, where high-functioning individuals have been found to pass traditional mentalising tasks but to show difficulties with more open-ended and naturalistic measures (Heavey et al. 2000). It is therefore important to try to replicate these findings in more naturalistic environments, which can often be much more stimulating and interpersonally demanding. One way forward may be through the use of virtual reality paradigms.

## Conclusion

The present study found that those with high AQ traits were overall less pro-social and sympathetic towards characters in need of their help, regardless of the strength of the social rule underpinning this. However, individuals with high AQ traits were equally able to estimate characters’ expectations for their help. This apparent conflict may reflect a reliance on social knowledge to estimate characters’ expectations on the basis of learned societal expectations, amongst other possible contributing factors. In line with this, those with high AQ traits tended to produce more rule-bound rationales to reason about why they were expected to help characters in the clear-cut condition, although not in the ambiguous condition where the social rules were less salient. This may again reflect a level of understanding that is more reliant on social knowledge, at the expense of utilising socio-emotional processes to engage directly with the characters’ needs. This reinforces and extends the findings of a previous study (Jameel et al. 2014), which indicated that those with high AQ traits demonstrated a relatively intact ability to assess characters’ perspectives in the context of reduced pro-social behaviour, and reported less personal reward for helping them.

Overall, the current study suggests that certain aspects of social judgement may be intact in those with high AQ traits, perhaps as a result of reliance on knowledge previously acquired through everyday experience in the social world. However, whilst reliance upon social knowledge may be useful for navigating social situations, it may be insufficient for motivating identification with others’ needs and subsequent pro-social behaviour. Although there is a wealth of literature exploring cognitive accounts of ASD, there is very little work either in those with high AQ traits or with ASD examining how these differences translate into everyday social functioning and impact on broader aspects of social interaction. Tasks such as that used in the present AQ study could be instrumental in providing a basis for a sensitive methodology to improve understanding of the nature of everyday difficulties associated with autistic characteristics, and could potentially be applied to clinical populations to improve interventions such as social-skill training.
